# The effect of policies regulating tobacco consumption on smoking initiation and cessation in Spain: is it equal across socioeconomic groups?

**DOI:** 10.1186/s12971-016-0109-4

**Published:** 2017-01-28

**Authors:** Jaime Pinilla, Ignacio Abásolo

**Affiliations:** 1 Departamento de Métodos Cuantitativos en Economía y Gestión (Universidad de Las Palmas de Gran Canaria). Facultad de Economía, Empresa y Turismo. Campus Universitario de Tafira, 35017 Las Palmas de Gran Canaria, Spain; 2 Departamento de Economía Aplicada y Métodos Cuantitativos; Instituto Universitario de Desarrollo Regional (Universidad de La Laguna). Facultad de Economía, Empresa y Turismo. Universidad de La Laguna, Campus de Guajara, 38071 La Laguna, Tenerife Spain

**Keywords:** Tobacco regulation, Socioeconomic groups, Smoking initiation and cessation, Smoking bans, National health survey, Spain

## Abstract

**Background:**

In Spain, the Law 28/2005, which came into effect on January 2006, was a turning point in smoking regulation and prevention, serving as a guarantee for the progress of future strategies in the direction marked by international organizations. It is expected that this regulatory policy should benefit relatively more to lower socioeconomic groups, thus contributing to a reduction in socioeconomic health inequalities. This research analyzes the effect of tobacco regulation in Spain, under Law 28/2005, on the initiation and cessation of tobacco consumption, and whether this effect has been unequal across distinct socioeconomic levels.

**Methods:**

Micro-data from the National Health Survey in its 2006 and 2011 editions are used (study numbers: 4382 and 5389 respectively; inventory of statistical operations (ISO) code: 54009), with a sample size of approximately 24,000 households divided into 2,000 census areas. This allows individuals’ tobacco consumption records to be reconstructed over five years before the initiation of each survey, as well as identifying those individuals that started or stopped smoking. The methodology is based on “time to event analysis”. Cox’s proportional hazard models are adapted to show the effects of a set of explanatory variables on the conditional probability of change in tobacco consumption: initiation as a daily smoker by young people or the cessation of daily smoking by adults.

**Results:**

Initiation rates among young people went from 25% (95% confidence interval (CI), 23–27) to 19% (95% CI, 17–21) following the implementation of the Law, and the change in cessation rates among smokers was even greater, with rates increasing from 12% (95% CI, 11–13) to 20% (95% CI, 19–21). However, this effect has not been equal by socioeconomic groups as shown by relative risks. Before the regulation policy, social class was not a statistically significant factor in the initiation of daily smoking (*p* > 0.05); however, following the implementation of the Law, young people belonging to social classes IV-V and VI had a relative risk of starting smoking 63% (*p* = 0.03) and 82% (*p* = 0.02) higher than young people of higher social classes I-II. On the other hand, lower social class also means a lower probability of smoking cessation; however, the relative risk of cessation for a smoker belonging to a household of social class VI (compared to classes I-II) went from 24% (*p* < 0.001) lower before the Law to 33% (*p* < 0.001) lower following the law’s implementation.

**Conclusion:**

Law 28/2005 has been effective, as after its promulgation there has been a decrease in the rate of smoking initiation among young people and an increase in the rate of cessation among adult smokers. However, this effect has not been equal by socioeconomic groups, favoring relatively more to those individuals belonging to higher social classes.

## Background

Tobacco use is the second global risk factor for mortality in the world, responsible of 9% of deaths globally −18% of deaths in high income countries- (and causing 71% of lung cancer deaths), just preceded by high blood pressure, a traditional risk factor for coronary artery disease, which causes 13% of deaths globally [1]. Prevalence of current tobacco smoking is therefore an important predictor of the future burden of tobacco related diseases and the social costs associated with it.^1^ Tobacco control policies in high income countries have succeed in reducing the exposure to this risk and ultimately in reducing the levels of diseases caused by tobacco.

The first policies to confront the phenomenon of smoking in Spain were adopted in the mid-1980s. The first action was to explicitly consider tobacco as a drug and include it as a substance subject to the policies and controls of the National Drug Plan 1985. Policies began by regulating tobacco advertising on billboards and institutional media, a ban on television, and the appearance of the first educational programs in schools and early interventions in the primary care and public health system [2]. In 2003, following the recommendations of the World Health Organization and European Directives, the first National Plan for Prevention and Control of Tobacco Use (NPPCT) was elaborated. Spain began to consider specific regulations on the control and prevention of smoking. The main objective of the NPPCT was to achieve harmonization between the different levels of public administration to avoid dispersion of competencies, and an insufficient systematic dissemination of action. It also aimed to make best use of the limited resources available and monitor more effectively compliance with regulations on sale, consumption and advertising of tobacco. However, for years the NPPCT could not prevent the Spanish legislation from continuing to occupy the lowest positions of the European Union with regard to the effectiveness of their strategies for smoking prevention [3].

In Spain, the Law 28/2005 (Law 28 hereinafter), which came into effect on January 2, 2006, was a turning point in smoking regulation and prevention, serving as a guarantee for the progress of future strategies in the direction marked by international organizations. Particularly, this Law was implemented setting out policies against smoking and the control, sale, supply, consumption and publicity of tobacco; the main aim of this law being the prevention and control of tobacco consumption.^2^

It is well known that mortality rates are higher among lower socioeconomic classes [4, 5]. There is also greater prevalence of smoking in individuals from lower socioeconomic levels, and some studies affirm that this is one of the main causes of the different mortality rates among socioeconomic groups [6, 7]. Furthermore, socioeconomic inequalities in the prevalence of smoking over time, rather than reducing or remaining stable, have become more accentuated. Nagelhout et al. in a study carried out in Holland over the period 2001–08 found evidence of an increase in socioeconomic inequalities in the prevalence of smoking, though only among women [8]. Bosdriez et al. researching the evolution of socioeconomic inequalities in the cessation of tobacco consumption in eleven European countries (including Spain) showed that inequalities had also increased significantly in the period 1987–2012 [9]. Moreover, Alves et al. in a study for Portugal, carried out between 1987 and 2006 found evidence of an increase in socioeconomic inequalities in the prevalence of smoking [10].

Given the aim of reducing socioeconomic inequalities in health^3^ and also given the higher prevalence of smokers (and other health risky behaviour) among lower social classes [6, 7], it is expected that policies that aim to control tobacco consumption should benefit relatively more to lower socioeconomic groups, thus contributing to a reduction in socioeconomic health inequalities. Is this what has happened in Spain after the promulgation of Law 28? The answer to this question is the main motivation for the aim of this paper, particularly, to analyze if the implementation and development of Law 28 has had an equal effect on different socioeconomic groups. Previous approaches that have explored the effect by socioeconomic level on policies of tobacco reduction can be classified into at least two groups: fiscal policies that affect the price of tobacco and regulation policies that control consumption through other interventions.

Regarding policies that affect tobacco prices (through tobacco tax changes), the results show, in general, that the effect of price changes is directly related to consumers’ income levels and purchasing power. In a pioneering study with data from surveys of British households for the period 1972–1990, Townsend et al. showed evidence of greater price elasticity of cigarettes among lower socioeconomic groups [11]. Subsequently, Farrelly et al. with data from the national health survey of the United States (collating data for 14 years, from 1976 to 1993) concluded that adults with lower than average incomes have a response four times more sensitive to changes in tobacco prices than those that have an above average income [12]. Gruber et al., in an analysis of tobacco expenditure, using data from a Canadian survey on family expenditure (1982 to 1998), found demand elasticity much higher for the lowest income quartile compared to families with higher incomes [13]. Along similar lines, Colman and Remler and Lee et al. also demonstrated that greater price elasticity is found in the decision to start smoking in lower income households compared with those with medium to high incomes [14, 15]. Additionally, the effects of price increases are greater among younger people and less well-off social groups [16]. The most recent estimations on price elasticities on cigarette demand can be found in studies that exploit, in different countries, the ITC survey model (International Tobacco Control): Nargis et al. for Bangladesh [17], Huang et al. and Yao et al. in China [18, 19], Cornelius et al. in the United States and Cowie et al. in Australia [20, 21]. The results obtained for these countries once again demonstrate that cigarette consumption among people from lower level socioeconomic groups is more sensitive to price variations than consumption among higher socioeconomic groups. Clearly, taxes on tobacco, despite being regressive from the economic viewpoint, are a useful tool to reduce the socioeconomic gradient of tobacco consumption.

As for regulatory policies to reduce tobacco consumption, the literature encompasses various interventions ranging from smoking prohibitions at work or in public spaces, to advertising restrictions for tobacco publicity and health warnings on cigarette packets or restrictions on access to cigarette vending machines. The literature also highlights the impact of these interventions by socioeconomic status of individuals (e.g., see the systematic review conducted by Thomas et al.) [22]. In studies analyzing the *acceptance* of policies for smoking restrictions, differences by socioeconomic levels were found. It was shown that the higher the income level, the higher the probability of accepting restrictions on the consumption of tobacco in bars/cafés [23]. These authors also found that the educational level of individuals was also positively related to acceptance of the ban on tobacco consumption in bars and cafés. Overall, therefore, individuals with higher education better understand the health risks posed by tobacco consumption, and accept them to a greater extent than the less educated. However, despite the educational level facilitates understanding of the warnings, it is not decisive for such comprehension to lead to cessation. Actually, studies with samples at different periods and interviews ‘before and after’ entry into force of smoking bans do not find any differences by socioeconomic level in the effectiveness of restrictions on consumption. This is the case of Becker et al., who studied the effect of a smoking ban in a hospital at USA, with survey data obtained six months before and six months after the implementation of the ban; they found that this policy was very effective but there were not found differences by occupation or education level [24]. Also, Willemsen, in a cross-sectional study with data from a survey from Dutch adult smokers, found no differences in smoking by educational level (low, medium or high) after the introduction of new health warnings [25]. Schaap et al. analysed the impact of tobacco control policies on quit ratios in 18 European countries (including Spain) by means of cross-sectional data from national health surveys; they show that both high and low educated smokers benefit equally from the nationwide tobacco control policies [26]. Finally, in their review, Thomas et al. neither find strong evidence of a socioeconomic gradient in the application of smoking restrictions and health warnings [22]. In short, at least compared to fiscal policies, there is no evidence that different public interventions in the field of tobacco regulation have a differential effect by socioeconomic level (i.e., favoring the lower socioeconomic groups).

This paper is divided into the following sections. In section 2, materials and method are presented. Section 3 contains the results and section 4 ends with the discussion of this research.

## Methods

### Data

In our study, we use microdata from the Encuesta Nacional de Salud or ENS (Spanish National Health Survey) in 2006–2007 (ENS06) and 2011–2012 (ENS11). The questionnaire provides past and present information on individuals’ tobacco consumption. At the time of the survey, individuals identified themselves as: daily/occasional smokers, ex-smokers or individuals that have never smoked. The questions “how long ago did you give up smoking?” and “what age did you start to smoke at?”, combined with the age at the time of the survey, allow us to reconstruct the smoking history of individuals over the five years prior to the beginning of the survey: between 2001 and 2005, with the responses from 2007 questionnaire (ENS06) and between 2006 and 2010, with the responses from 2012 (ENS11).

**Fig. 1:**
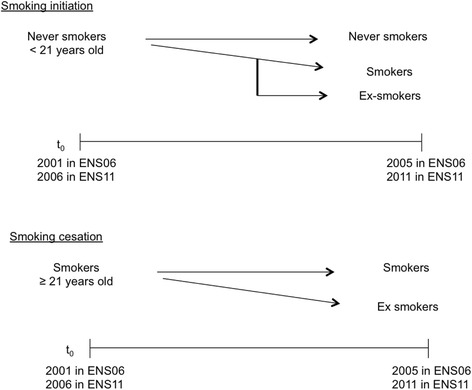
Diagram of transitions between cohorts. Smoking initiation and cessation

Our analysis attempts to detect the presence of socioeconomic inequalities in the process of tobacco consumption initiation and cessation. In particular, we focus on initiation of smoking by young people under 21 years old and cessation by regular smokers of 21 years old or older. We use the threshold of 21 years old as the initiation time t_0_, because we consider this age as the minimum age in which individuals adopt fixed habits of daily smoking [27, 28]. The comparison of the results between cohorts ENS06 and ENS11 allows us to evaluate the effect of Law 28. Figure 1 depicts the ages, transitions and years of the cohorts.

The ENS has, in its editions ENS06 and ENS11, a sample size of 29,478 and 21,508 individuals, respectively. For the analysis of the initiation of tobacco consumption among young people, we have a sample of 3,412 observations, of which 1,991 correspond to ENS06 and 1,421 to ENS11. In the analysis of smoking cessation, the total sample consists of 13,207 individuals, with 7,567 and 5,640 individuals from ENS06 and ENS11, respectively.

Both ENS surveys provide information on socioeconomic status (occupation and educational level) and other demographic variables (gender or region of residence) and about the state of health of interviewees, which we also consider in this study.

After four years of its application, Law 42/2010 was passed amending Law 28/2005 and extending the smoking ban to all enclosed spaces. The effect of this new law has not been evaluated in our work in the absence of an appropriate database. In 2014, the ENS was replaced by the European Health Survey (EHS). The fieldwork of the EHS14 was conducted between January 2013 and February 2014. The previous dates leave us a margin of just two years to analyze changes with respect to smoking. The lack of information on the date and time without smoking in ex-smokers prevents us from analyzing the process of abandonment.

### Variables

The presence of socioeconomic inequalities are studied using the “social class” variable of the reference person in the household, which we group into four categories based on the National Classification of Occupations: class I-II (Directors and managers with university degrees), class III (intermediate professions and self-employed), classes IV and V (skilled and partly-skilled occupations), and class VI (unskilled workers).

In the analysis of initiation in tobacco consumption, we incorporate as control variables as well as gender, the fact that some individuals reside in areas where there is low tax on tobacco (i.e., the Canary Islands and Ceuta or Melilla). The objective is to capture any possible price effects, as living in these areas mean tobacco is sold at substantially lower prices than in the rest of Spain. As for the analysis of tobacco cessation, we add, apart from gender and residence in a low-tobacco duty area, the level of studies of interviewees. This level is divided into four groups: primary education and below, secondary, vocational training or baccalaureate and university level. In addition, other work-related variables (if the individual works or is unemployed) and health variables (if the interviewee has suffered a heart attack), are included. Regarding work, Law 28 bans smoking in the workplace, which means that those in work have an incentive to cut down or give up smoking [29]. As for health, suffering a high mortality illness, like a heart attack, is an important incentive to stop smoking [30, 31]. Age could not be included in any of the models as this changes over time, the same occurs with level of studies among youngsters in the model for initiation of smoking. The income variable, measured by the ENS as average household income, is not included in the model as a socioeconomic indicator for two key reasons: on the one hand, the values of reference intervals between the two surveys are very different, and on the other, this variable has large number of observations missing.

### Statistical analysis

To obtain the maximum possible information of the sequence of change (starting and giving up) and the moment in time when they happen, we opted for a methodology involving “time to event analysis” instead of transversal analysis with models of discrete choice.

We adjust Cox’s proportional hazard models, where we measure the effects of a set of explanatory variables on the conditional probability of change, either initiation of regular smoking or cessation (ex-smoker).

Cox’s regression model is determined by the relationship:

$$ h\left( t/ x\right) = {h}_0(t) \exp \left( X\hbox{'}\beta \right) $$

where temporal/time dependence is included in the rate of base risk *h*_*0*_ *(t)*, and the explanatory variables act in log-linear form, *exp(X'β)*, where *β* is a vector of unknown regression coefficients that parameterizes the model.

Owing to the existence of incomplete data –right-censored data values-, the parameters of Cox’s model are estimated by partial likelihood, allowing consistent estimates to be obtained [32–34].

The proportionality of the explanatory variables was checked both graphically as well as statistically through the analysis of Schoenfeld residuals [35]. Explanatory variables incorporate step-by-step models with the aim of detecting possible effects of multi-colinearity. As a statistical test of best fit, the verisimilitude ratio test is used. The analysis was carried out with the statistical package Stata (version 13.1, Texas: College Station; 2013).

## Results

Table 1 shows the descriptive statistics of the variables that are included in the model for initiating tobacco consumption. With respect to the cohort 2001–2005, in 2001 there were 1,991 youngsters under the age of 21 years-old who had never smoked. Between 2001 and 2005, 1,492 young people remained as non-smokers, but 499 (25%) began smoking regularly. The average age of these young people at the time of the survey was 20.4 years old, with 52% being women, 6% residing in the Canary Islands or, Ceuta and Melilla and the majority belonging to the social class IV-V. Regarding the 2006–2010 cohort, in 2006 there were a total of 1,421 youngsters who were non-smokers. Between 2006 and 2010, 1,153 (81%) remained non-smokers, and 268 (19% remaining) initiated smoking as a daily habit. The average age of these youngsters at the time of the survey was 20.5 years old, with 51% being female, and 7% residents in the Canary Islands, Ceuta or Melilla and the majority, 47%, belonging to social classes IV-V.

**Table 1:** Description of the under 21 year-old cohorts that initiated daily, 2001–2005 and 2006-2010

Dependent Variables	ENS06 period 2001–2005 *N* = 1991		ENS11 period 2006–2010 *N* = 1421	
Initiation as a daily smoker	Proportion (IC 95%)	Observations	Proportion (IC 95%)	Observations
Yes	0.25 (0.23;0.27)	499	0.19 (0.17;0.21)	268
No	0.75 (0.73;0.77)	1492	0.81 (0.79;0.83)	1153
Pearson chi2(1) = 18.31 Pr = 0.000				
Independent variables at the time of the survey	Average proportion	Observations	Average proportion	Observations
Average age	20.38	1991	20.50	1421
Women	0.52	1991	0.51	1421
Social Class:				
Class I-II	0.19	377	0.18	238
Class III	0.23	449	0.17	234
Class IV-V	0.43	844	0.47	631
Class VI	0.14	275	0.18	238
Resident in the Canary Islands, Ceuta or Melilla	0.06	1991	0.07	1421

Table 2 provides the statistics of the variables involved in the model of cessation of tobacco consumption. Regarding the cohort of 2001–2005, in 2001 there were 7,567 adults over 21 years old who were daily smokers. Between 2001 and 2005 6,650 (88%) remained as daily smokers and 917 (12%) stopped smoking. The average age at the time of the survey was 43.6 years old, 50% were women and 6% were resident in the Canary Islands, Ceuta or Melilla. There were 62% who were employed, 1.3% had suffered a heart attack at some time, 32% had secondary level studies and the majority, 44% belonged to social classes IV-V. As for the 2006–2010 cohort, in 2006 there were 5,640 daily adult smokers. Between 2006 and 2010, 4,516 (80%) remained daily smokers, and 1,124 (20%) stopped smoking. The average age at the time of the survey was 45.5 years old, with 43% being women, 6% residents in the Canary Islands, Ceuta or Melilla, 55% in employment, 1.7% had a heart attack, 48% with secondary level studies and the majority, 50%, belong to social classes IV-V.

**Table 2:** Description of adult cohorts over the age of 21 years old that ceased smoking, 2001–2005 and 2006–2010

Dependent Variable	ENS06 period 2001–2005 *N* = 7567		ENS11 period 2006–2010 *N* = 5064	
Ex-smoker	Proportion (IC 95%)	Observations	Proportion (IC 95%)	Observations
Yes	0.12 (0.11;0.13)	917	0.20 (0.19;0.21)	1124
No	0.88 (0.87;0.89)	6650	0.80 (0.79;0.81)	4516
Pearson chi2(1) = 150.88 Pr = 0.000				
Independent Variables at the time of the survey	Average proportion	Observations	Average proportion	Observations
Average age	43.64	7567	45.47	5640
Women	0.50	7567	0.43	5640
Social Class:				
Class I-II	0.19	1423	0.17	958
Class III	0.24	1804	0.19	1055
Class IV-V	0.44	3286	0.50	2764
Class VI	0.13	951	0.13	749
Level of education:				
Primary or lower	0.18	2876	0.16	926
Secondary	0.32	1680	0.39	2180
Baccalaureate or vocational	0.23	1764	0.23	1317
University	0.16	1207	0.22	1217
Employed	0.62	7552	0.55	5063
Suffered heart attack	0.01	7567	0.02	5063
Resident in the Canary Islands, Ceuta or Melilla	0.06	7567	0.06	5063

**Fig. 2:**
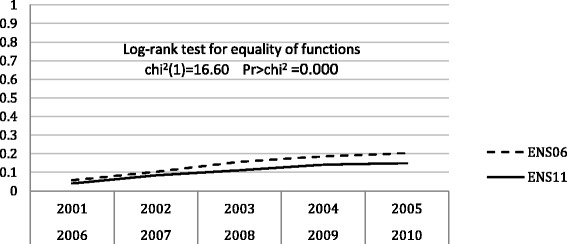
Kaplan-Meier failure function for initiation of smoking

**Fig. 3:**
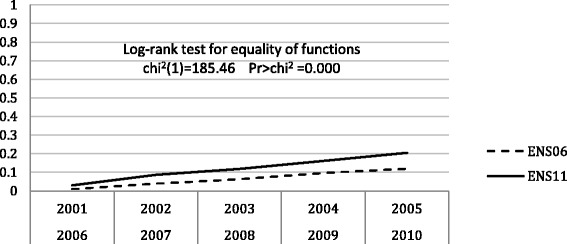
Kaplan-Meier failure function for cessation of smoking

Figures 2 and 3 show the Kaplan-Meier failure functions for initiation and cessation of smoking. In both models, the test log-rank for equality of functions rejects the equality hypothesis, consequently both cohorts 2001–2005 and 2006–2010 depict different transitions at the initiation and cessation. The estimation of the models with explanatory variables is performed for each cohort separately.

In Table 3, the results from the estimations obtained from Cox’s proportional hazard models. The coefficients are presented as hazard ratios (also called relative risks). Regarding the influence of socioeconomic level in initiation of daily smoking by under 21 year-olds, the social class was not statistically significant in the 2001–2005 cohort, which was the cohort prior to Law 28. In the 2006–2010 cohort, social class has a significant effect on the risk of becoming a daily smoker. Young people that belong to social classes IV-V (skilled and partially skilled workers) and VI (unskilled workers) show a 63 to 82% higher relative risk, respectively, of initiating daily smoking than the more well-off social classes I-II (directors and managers with university degrees).

**Table 3:** Cox estimations of proportional randomness on the initiation and cessation of tobacco consumption: Cohorts 2001–2005 and 2006–2010

Explanatory Variables	Initiation		Cessation	
	ENS06 2001–2005	ENS11 2006–2010	ENS06 2001–2005	ENS11 2006–2010
	Hazard ratio (std. err.)		Hazard ratio (std. err.)	
Female	1.10 (0.11)	0.81 (0.12)	1.11 (0.76)	0.88*(0.06)
Social class:				
Class I-II (reference)				
Class III	0.87 (0.14)	1.42 (0.38)	0.93 (0.09)	1.08 (0.10)
Class IV-V	1.09 (0.15)	1.63* (0.37)	0.88 (0.09)	0.85 (0.08)
Class VI	0.94 (0.17)	1.82* (0.47)	0.76**(0.10)	0.67**(0.09)
Resident in the Canary Islands, Ceuta or Melilla	0.73 (0.17)	0.62 (0.18)	0.86 (0.13)	0.82 (0.11)
Working			0.83**(0.06)	0.94 (0.06)
Has suffered an heart attack			3.10**(0.57)	2.87**(0.45)
Level of studies:				
Primary or below (reference)				
Secondary			1.15 (0.11)	0.78*(0.10)
High School or vocational train.			1.26**(0.12)	0.87 (0.13)
University students			1.49**(0.16)	1.08 (0.15)
Num. Observations	1836	1281	7426	5526
Log pseudolikelihood	−2822.08	−1355.25	−7803.19	−9315.31

* (*p*-value < 0.05) ** (*p*-value < 0.01)

The analysis of cessation of daily smoking by adults showed that the lower the social class, the lower the probability of stopping smoking. For cohorts 2001–2005 and 2006–2010, belonging to social class VI household leads to a 24 and 33% lower risk of smoking cessation compared to households from classes I_II, respectively. There is also a greater probability of tobacco cessation among individuals with higher education levels (higher vocational or university studies) than those with lower educational levels, although these differences were only statistically significant for the 2001–2005 cohort.

Regarding gender, being a woman was not statistically significant in either of the models, except in the cessation model for the 2006–2010 cohort. In this cohort, the relative risk of stopping smoking was 12% lower in women compared to men. Suffering a heart attack significantly increased the probability of cessation. By contrast, residing in a lower tobacco tax zone was not shown to have any statistically significant effect. Finally, being employed and in the 2001–2005 cohort meant a reduction in the relative risk of stopping smoking of 17%, though this situation was not statistically significant in the 2006–2010 cohort.

## Discussion

Our results show that the battery of smoking regulation policies that have been adopted in Spain between 2006 and 2011 with the development of Law 28, have coincided with an increase in tobacco cessation rates in adults and with a reduction in the likelihood of youth and adolescents to start as daily smokers. These results are consistent, therefore, with the idea that regulatory policies have been effective. However, this effectiveness has been unequal across social classes, benefitting (in terms of lower proportion of smokers) individuals belonging to higher social class households. Regarding possible explanations, some previous evidence suggests that lower socioeconomic status is associated with less understanding of the harmful health effects of tobacco consumption [36]. In addition, some studies have found increased nicotine dependence in individuals of lower socioeconomic status, hampering their chances of cessation [37]. However, our results go in the opposite direction to some previous research [22, 24–26], which find no differences in smoking by educational level after the introduction smoking bans.

Law 28 includes a series of measures that promote smoking prevention actions through health education and information. However, there is no reference in the Law to the implementation of specific programs for different socioeconomic levels to help reduce such health inequalities. The evidence found in this investigation -i.e., that Law 28 has been less effective for lower socioeconomic groups, indicates that part of its potential has been undermined, not in reducing the prevalence of smoking but in reducing socioeconomic inequalities in health that have smoking as their leading cause. If socioeconomic inequalities want to be reduced through effective regulations on tobacco consumption, preventive actions should be directed more strongly at young people and adults belonging to lower social classes. Likewise with gender: though in the period 2001–2005 there was no significant difference in the cessation (or initiation) by gender, in the period 2006–2011 it was noted that the relative risk of cessation in women was lower, indicating that policies controlling smoking have affected men relatively more. Educational and health actions should, therefore, impact more on women, leading to a relative improvement in their health and that of the fetus, in the case of pregnant women.

Our research is not exempt from some limitations. First, the smoking history of each individual has been reconstructed from his/her memories at the time of the survey with the potential bias that individual memory may imply. Second, in some explanatory variables taken from the questionnaire, the information prevents the historical evolution of individuals from being reconstructed. This is the case for educational level, employment status, social class and place of residence; however, with regard to the social class of the household-the key variable to identifying socioeconomic inequalities is based on occupations that the reference person has or has had and is not expected to change in the 5-year intervals. Third, this research fails to address an important effect, which is to measure the reduction in the level of tobacco consumption. Questions used in the questionnaire available from the ENS (if consumption at the time of the survey is greater than, equal to or less than two years) make the analysis of this situation within the framework of the duration models impossible. Finally, as noted in the introduction section, during the period analyzed in this research, in addition to regulatory policy (through the development of Law 28), there have been successive increases in tobacco taxes. Thus, it cannot be excluded that our results are affected by this tax factor. However if -as evidence in the related literature shows [11–16]- price elasticity of tobacco demand is inversely related to socioeconomic status, then, the relatively lower effectiveness of regulatory policies on lower social classes resulting from our study, would have been underestimated (i.e., socioeconomic inequality in the effectiveness of regulatory policies would be even greater).

## Conclusion

The smoking regulation contained in the Law 28/2005 has been effective, as after its promulgation there has been a decrease in the rate of smoking initiation among young people and an increase in the rate of cessation among adult smokers. Specifically, the initiation rates among young people went from 25 to 19% following the implementation of the Law, and the change in cessation rates among smokers was even greater, with rates increasing from 12 to 20%. However, this effect has not been equal by socioeconomic groups as shown by relative risks. Before the regulation policy, social class was not a statistically significant factor in the initiation of daily smoking; however, following the implementation of the Law, young people belonging to social classes IV-V and VI had a relative risk of starting smoking 63 and 82% higher than young people of higher social classes I-II. On the other hand, lower social class also means a lower probability of smoking cessation; however, the relative risk of cessation for a smoker belonging to a household of social class VI (compared to classes I-II) went from 24% lower before the Law to 33% lower following the law’s implementation.

## Abbreviations

EHS: European health survey
ENS: Spanish national health survey
ENS06: 2006–07 Spanish national health survey
ENS11: 2011–12 Spanish national health survey
ITC: International tobacco control
NPPCT: National plan for prevention and control of tobacco use

## Footnotes

1 In Spain, prevalence of smoking among adults aged ≥15 years in 2012 is 33,7% for men and 28.0% for women, above the international average, particularly in the case of women (average of 9.1%), not so much for men (33,6%) [38].

2 Together with Law 28/2005, there was a royal decree that modified tax rates on tobacco, further modified in February and October of the same year and also in subsequent years.

3 In Spain, the concern to reduce social health inequalities can be shown in the National Health System Laws (The General Health Act, 1986 and the National Health System Cohesion and Quality Act, 2003) and is also sustained by opinions of the general public [39].

## References

[1] World Health Organization. Global Health Risks. Mortality and burden of disease attributable to selected major risks. 2009. http://www.who.int/healthinfo/global_burden_disease/GlobalHealthRisks_report_full.pdf.

[2] Cuesta-Arzamendi JL, Muñagorri I, Arana X. Políticas y legislación en materia de tabaco. Informe (Observatorio Vasco de Drogodependencias) n°20; Vitoria-Gasteiz: Eusko Jaurlaritzaren Argitalpen Zerbitzu Nagusia. Servicio Central de Publicaciones del Gobierno Vasco. 2009.

[3] Hyland A, Barnoya J, Corral JE (2012). Smoke-free air policies: past, present and future. Tob Control.

[4] Mackenbach JP, Stirbu I, Roskam AJ, Schaap MM, Menvielle G, Leinsalu M (2008). Socioeconomic inequalities in health in 22 European countries [research support, non-U.S. gov’t]. N Engl J Med.

[5] Mackenbach JP (2012). The persistence of health inequalities in modern welfare states: the explanation of a paradox. Soc Sci Med.

[6] Eikemo TA, Hoffmann R, Kulik MC, Kulhánová I, Toch-Marquardt M, Menvielle G (2014). How can inequalities in mortality be reduced? a quantitative analysis of 6 risk factors in 21 European populations. EURO-GBD-SE Consortium. PLoS One.

[7] Mackenbach JP, Kulhánová I, Bopp M, Deboosere P, Eikemo TA, Hoffmann R (2015). Variations in the relation between education and cause-specific mortality in 19 European populations: a test of the “fundamental causes” theory of social inequalities in health. EURO-GBD-SE Consortium. Soc Sci Med.

[8] Nagelhout GE, De Boer D, Kunst AE, Van der Meer R, De Vries H, Van Gelder BM (2012). Trends in socioeconomic inequalities in smoking prevalence, consumption, initiation, and cessation between 2001 and 2008 in the Netherlands. Findings from a national population survey. BMC Public Health.

[9] Bosdriesz JR, Willemsen MC, Stronks K, Kunst AE (2015). Socioeconomic inequalities in smoking cessation in 11 European countries from 1987 to 2012. J Epidemiol Community Health.

[10] Alves J, Kunst AE, Perelman J (2015). Evolution of socioeconomic inequalities in smoking: results from the Portuguese national health interview surveys. BMC Public Health.

[11] Townsend J, Roderick P, Cooper J (1994). Cigarrete smoking by socioeconomic group, sex and age: effects of price, income and health publicity. Br Med J.

[12] Farrelly MC, Bray JW, Pechacek T, Woollery T (2001). Response by adults to increases in cigarette prices by sociodemographic characteristics. South Econ J.

[13] Gruber J, Sen A, Stabile M. Estimating price elasticities when there is smuggling: the sensitivity of smoking to price in Canada. Journal of Health Economics. 2003;821–42. 10.1016/S0167-6296(03)00058-4 12946461

[14] Colman G, Remler DK. Vertical equity consequences of very high cigarette tax increases: if the poor are the ones smoking, how could cigarette tax Increases be progressive?. NBER Work Pap Ser 10906. 2004.

[15] Lee JM, Hwang TC, Ye CY, Chen SH. The effect of cigarette price increase on the cigarette consumption in Taiwan: evidence from the national health interview surveys on cigarette consumption. BMC Public Health. 2004;4:61. doi:10.1186/1471-2458-4-61. 10.1186/1471-2458-4-61 PMC539350 15598345

[16] Levy DT, Chaloupka F, Gitchell J (2004). The effects of tobacco control policies on smoking-rates: a tobacco control scorecard. J Public Health Manag Pract.

[17] Nargis N, Ruthbah UH, Hussain AKM (2014). The price sensitivity of cigarette consumption in Bangladesh: evidence from the International Tobacco Control (ITC) Bangladesh Wave 1 (2009) and Wave 2 (2010) Surveys. Tob Control.

[18] Huang J, Zheng R, Chaloupka F (2014). Chinese smokers’ cigarette purchase behaviours, cigarette prices and consumption in China: findings from the ITC China survey. Tob Control.

[19] Yao T, Huang J, Sung HY (2014). Who purchases cigarettes from cheaper sources in China? Findings from the ITC China survey. Tob Control.

[20] Cornelius ME, Driezen P, Fong GT (2014). Trends in the use of premium and discount cigarette brands: findings from the ITC US surveys (2002–2010). Tob Control.

[21] Cowie GA, Swift E, Borland R (2014). Cigarette brand loyalty in Australia: findings from the ITC four country survey. Tob Control.

[22] Thomas S, Fayter D, Misso K, Ogilvie D, Petticrew M, Sowden A (2008). Population tobacco control interventions and their effects on social inequalities in smoking: systematic review. Tob Control.

[23] Tang H, Cowling DW, Lloyd JC, Rogers T, Koumjian KL, Stevens CM (2003). Changes of attitudes and patronage behaviors in response to a smoke-free bar law. Am J Public Health.

[24] Becker D, Conner H, Waranch H, Stillman F, Pennington L, Lees P (1989). The impact of a total ban on smoking in the Johns Hopkins children’s center. JAMA.

[25] Willemsen MC (2005). The new EU cigarette health warnings benefit smokers who want to quit the habit: results from the Dutch Continuous Survey of Smoking Habits. Eur J Public Health.

[26] Schaap MM, Kunst AE, Leinsalu M, Regidor E, Ekholm O, Dzurova D (2008). Effect of nationwide tobacco control policies on smoking cessation in high and low educated groups in 18 European countries. Tob Control.

[27] Caballero-Hidalgo A, González B, Pinilla J, Barber P (2005). Factores predictores del inicio y consolidación del consumo de tabaco en adolescentes. Gac Sanit.

[28] Hammond D (2005). Smoking behaviour among young adults: beyond youth prevention. Tob Control.

[29] Anger S, Kvasnicka M, Siedler T (2011). One last puff? Public smoking bans and smoking behavior. J Health Econ.

[30] Lightwood JM, Glantz SA (1997). Short-term economic and health benefits of smoking cessation: myocardial infarction and stroke. Circulation.

[31] Mähönen MS, McElduff P, Dobson AJ, Kuulasmaa KA, Evans AE (2004). Current smoking an the risk of non-fatal myocardial infarction in the Who MONICA project populations. Tob Control.

[32] Cox DR (1975). Partial likelihood. Biometrika.

[33] Tsiatis A (1981). A large sample study of Cox’s regresssion model. Ann Stat.

[34] Efron B (1977). The efficency of Cox’s likelihood function for censored data. J Am Stat Assoc.

[35] Harrell FE, Robert P (1986). The PHGLM procedure. SUGI supplemental library user’s guide.

[36] Hyland A, Borland R, Li Q, Yong HH, McNeill A, Fong GT (2006). Individual-level predictors of cessation behaviours among participants in the International Tobacco Control (ITC) four country survey. Tob Control.

[37] Siahpush M, McNeill A, Borland R, Fong GT, Siahpush M, McNeill A, Borland R, Fong GT (2006). Socioeconomic variations in nicotine dependence, self-efficacy, and intention to quit across four countries: findings from the International Tobacco Control (ITC) four country survey. Tob Control.

[38] World Health Organization. Health Statistics and Information System. 2015. http://www.who.int/healthinfo/en/.

[39] Abásolo I, Tsuchiya A (2013). Is more health always better for society? Exploring public preferences that violate monotonicity. Theory Decis.

